# Modelling a Response of Complex-Phase Steel at High Strain Rates

**DOI:** 10.3390/ma17102302

**Published:** 2024-05-13

**Authors:** Andrej Škrlec, Tadej Kocjan, Marko Nagode, Jernej Klemenc

**Affiliations:** Faculty of Mechanical Engineering, University of Ljubljana, 1000 Ljubljana, Slovenia; andrej.skrlec@fs.uni-lj.si (A.Š.); tadej.kocjan@fs.uni-lj.si (T.K.); marko.nagode@fs.uni-lj.si (M.N.)

**Keywords:** complex phase steel, Cowper–Symonds model, response surface, multi-criterion objective function, genetic algorithm

## Abstract

In this article, a response of the complex-phase high-strength steel SZBS800 was modelled by considering the strain-rate influence. The material’s response was first measured with a series of standard tensile tests at lower strain rates. Higher strain rates were achieved using the unconventional test of shooting the ball into flat specimens. A viscoplastic formulation of the Cowper–Symonds material model was applied to consider the strain-rate effects. The parameters *SIGY*, *p*, and *C* of the material model were estimated using a step-wise procedure. First, rough estimates of the three parameters were obtained from the tensile tests using the grid search method. Then, the parameters *p* and *C* were fine-tuned using the reverse engineering approach. With the help of explicit dynamic simulations and all the experimental data, a multi-criteria cost function was defined and applied to obtain a smooth response function for the parameters *p* and *C*. Its optimum was determined by a real-valued genetic algorithm. The optimal values of the estimated parameters model the material response well, although a domain of optimum candidates spans two orders of magnitude for the parameter *p* and a few orders of magnitude for the parameter *C*.

## 1. Introduction

To simulate the behaviour of a product under impact loading conditions, the applied material model should consider the strain-rate dependencies in the elastic–plastic domain. For this purpose, two material models are predominantly applied in practice: a Cowper–Symonds material model and a Johnson–Cook material model. The two material models basically differ according to the functional form of the strain-rate dependency. The strain-rate dependency of the yield stress in the Cowper–Symonds material model follows the power law equation. In contrast, this dependency is logarithmic in the Johnson–Cook material model. Another difference between the two material models is that the yield curve is always scaled according to the strain-rate influence in the Johnson–Cook model, but the Cowper–Symonds material model also allows for a vertical translation of the Yield curve as a function of the plastic strain rate [1,2].

The strain rate affects up to a few 10 s^−1^, which can be experimentally determined on universal testing machines. However, to reach strain rates of an order of magnitude 1000 s^−1^ or more, different experimental arrangements are needed. The most popular among them are a split Hopkinson bar test, a Taylor impact test, and a test of projectile shooting into a deformable body. While the parameters of the material models can be relatively simply determined from the tensile tests, this task becomes a challenge for the split Hopkinson bar test and the two impact tests. For this purpose, different reverse-engineering approaches can be followed [3,4]. Hernandez et al. [3] have proposed a dynamic characterisation computational procedure to determine the material parameters from a single Taylor impact test. The procedure involves the formulation and solution of an inverse problem to determine the Cowper–Symonds parameter of metals using the silhouette of a deformed Taylor specimen as input. Skrlec and Klemenc [4] have shown how the material parameters of the Johnson–Cook material model can be estimated from the projectile shooting test using the Taguchi design of experiments combined with explicit dynamic simulations, response surface for the cost function, and a numerical optimisation scheme.

In the past decade, the extensive development of advanced high-strength steels has been achieved for different purposes. The major user of such steels is the automotive industry, which has high product safety requirements. To use the full potential of such innovative steels, their material characteristics should be properly estimated. To achieve the requested crashworthiness of the road vehicles, the mechanical response of the crushable structural elements should be well predicted. This is only possible if the strain-rate-dependent material properties of such elements are known. Therefore, much research has recently been conducted related to the strain-rate-dependent behaviour of mild steels, advanced high-strength steels, and other high-strength alloys. Li et al. [5] investigated the behaviour of a Q390D steel at strain rates ranging from 0.0001 to 3000 s^−1^. Q390D had a significant rate of strain hardening. The yield and ultimate strength of the Q390D steel increased significantly with the increasing strain rate. The quasi-static strain rate was 0.00083 s^−1^. The strain-rate response was predicted by fitting the Cowper–Symonds model to the experimental data. Gupta et al. [6] studied the deformation behaviour and notch sensitivity of a super duplex stainless steel at different strain rates and temperatures. Qin et al. [7] investigated the mechanical behaviour of dual-phase high-strength steels (DP700 and DP500) under high strain rate tensile loading. The parameters of the Johnson–Cook material model were determined from experimental data well in the plastic zone. Digital image correlation was used with high-speed photography to study the strain localisation in the tensile specimens at high strain rates. Hu et al. [8] investigated the dynamic tensile characteristics of TRIP600, TRIP800, DP600, and DP800 steels at strain rates from 0.003 to 200 s^−1^. They obtained quantitative results for the increase in the flow stress as a function of the strain rate for both observed types of steel sheets. The results show that the DP-type steel sheets are more sensitive to the strain rate when compared to the TRIP-type steel sheets at the intermediate strain rates. They also concluded that the fracture elongation of the TRIP-type steel sheets decreases with increasing strain rates from 0.003 to 0.1 s^−1^ but then increases up to the strain rate of 100 s^−1^ due to the local strain rate hardening. The elongation of the DP-type steel sheets increases monotonically as the strain rate increases in contrast to the classical conjecture. Yu et al. [9] made quasi-static and dynamic tensile experiments for DP600 steel for the strain rate range between 10^−4^ and 10^3^ s^−1^ by using conventional testing apparatus and the BTIA test. The results show that an obvious strain-rate-dependent mechanical behaviour exists for DP600 steel. The yield stress values at high strain rates are nearly twice those at low strain rates. The yield mechanisms are also different at high and low strain rates. The upper and lower yield points obviously exist at high strain rates, which is different from the material response at low strain rates. Shang et al. [10] studied the strain-rate and stress-state-dependent ductile fracture model of a S690 high-strength steel. They discovered that the strain rate effect of high-strength steel at the range of 10^−3^–10^3^ s^−1^ is well described by the Cowper–Symonds strain-rate-hardening criterion. They also modified the Johnson–Cook constitutive model using the Cowper–Symonds strain-rate-hardening description. Yang et al. [11] investigated the strain-rate-dependent behaviour of the S690 high-strength structural steel at intermediate strain rates. The tests indicated that the strain rate had an obvious influence on the behaviour of S690 steel, i.e., both the yield stress and tensile strength increased remarkably with increasing strain rate. The test results were then used to determine the coefficients of two commonly used strain-rate-dependent material models, the Cowper–Symonds model and the Johnson–Cook model. Wang et al. [12] researched the dynamic deformation and fracture mechanisms of Ti6Al4V over a wide strain rate range from quasi-static up to 10^4^ s^−1^. The effects of the strain rate on the material deformation and associated fracture mechanism are discussed. The results demonstrate a strong strain-rate sensitivity of damage evolution of Ti6Al4V. Mahalle et al. [13] predicted static and dynamic flow stress behaviour of Inconel 718 alloy based on a modified Cowper–Symonds model, which was observed to provide better prediction in terms of statistical parameters for Inconel 718. Recently, studies with machine-learning-based modelling of strain rate and temperature effects were conducted [14,15].

This article deals with the strain-rate-dependent material properties of a high-strength steel SZBS800 (an EN 1.0998 equivalent steel, Salzgitter, Germany). This complex-phase steel has recently been used in some automotive applications, which should ensure proper passenger safety during a vehicle crash. It was determined from preliminary tensile experiments that the yield curve of the SZBS800 steel is shifted almost in parallel along the ordinate axis in the σ-ε diagram as a function of the strain rate. For this reason, it was decided to model the material characteristics of the SZBS800 steel with the Cowper–Symonds model using a viscoplastic formulation [1], which mimics such a material response. The parameters of the selected material model were estimated using the modified reverse engineering methodology of Skrlec and Klemenc [4]. Instead of applying the design of the experiment method, the rough estimates of the Cowper–Symonds strain-rate-dependent parameters were obtained from the tensile tests using a two-phase procedure. Afterwards, the material parameters were fine-tuned on the basis of low- and high-strain rate experiments. For the high-strain rate experiments, the test of shooting a steel ball into a deformable plate-like specimen was applied. The results of all the experiments were combined with the results of numerical simulations into a multi-criterion objective function. Its response surface was determined, and the optimal values for the Cowper–Symonds strain-rate-dependent parameters were estimated using a real-valued genetic algorithm.

The article is structured as follows. After the introductory section, the experiments are first explained. Then, the explicit dynamic simulations of the shooting test are presented. Next, the theoretical background is given for the applied multi-criteria objective function and its approximation with the response surface. In the third section, the experimental and numerical results are presented and discussed. The optimal estimates of the strain-rate-dependent parameters of the Cowper–Symonds model are presented with the results from the literature for similar steels. The article ends with a concluding section and a list of references.

## 2. Materials and Methods

### 2.1. Tensile Stress–Strain Curves at Different Low-Strain Rates

A methodology was applied to characterise the strain-rate-dependent material behaviour of high-strength steel with the commercial designation SZBS800 (EN 1.0998 steel, Salzgitter Flachstahl GmbH, Salzgitter, Germany). The chemical composition of the steel is presented in Table 1.

**Figure 1:**
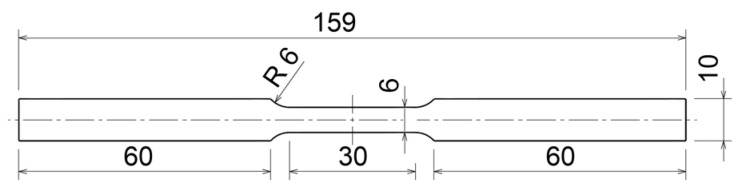
Flat specimen for tensile tests. Dimensions are in mm.

The low strain rate material properties (elastic modulus, yield stress, ultimate tensile stress) were measured according to the ASTM E8/E8M standard [17]. The specimen geometry with a gauge length of 25 mm was used, which is presented in Figure 1. To obtain the stress–strain curves of the observed material, 13 tensile tests were performed with a 100 kN MTS Landmark testing machine with controlled loading rates at different values: 0.0028 s^−1^, 0.028 s^−1^, 0.14 s^−1^, 2.5 s^−1^, 5.2, and 5.4 s^−1^. At the lowest strain rate, strains were measured with an MTS 834.11F-24 mechanical extensometer, MTS Systems, Eden Prairie, MN, USA. At the same time, strains were also determined using the digital image correlation (DIC) method. For this purpose, the speckled colour pattern on the specimen was recorded with an i-SPEED 508 high-speed camera from IX Cameras, Rochford, UK. The sampling rate varied according to the loading rate and reached 4096 frames per second at the highest loading rate of 5.4 s^−1^. The camera’s frame rate was synchronised with the data acquisition sampling rate of the MTS testing machine. Digital image processing was carried out with Dantec Istra4D V4.10 software (https://www.dantecdynamics.com/new-istra4d-v4-10-software-release/, accessed on 30 April 2024). For the tests at higher strain rates, only the DIC method was used to estimate the strains.

**Figure 2:**
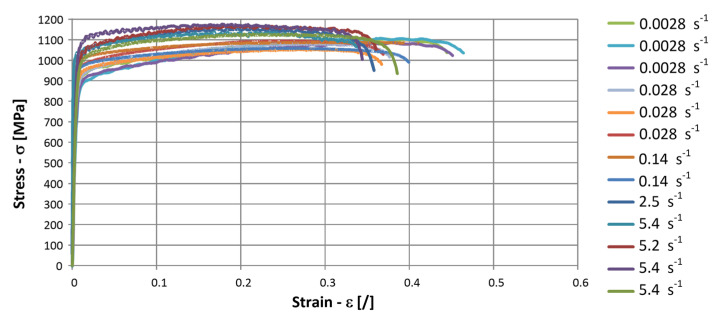
Measured stress-strain diagrams for the SZBS800 steel.

Measured stress–strain diagrams are presented in Figure 2. The average yield offset stress (Rp0.2) at the lowest strain rate and the ultimate tensile strength from the tensile tests were about 800 MPa and 1500 MPa, respectively.

The measured engineering stress–strain curves were first transformed into true stress–true strain curves according to the following equations [18,19]:

(1) $$\tilde{\varepsilon }=ln\left(1+\varepsilon \right)$$

(2) $$\tilde{\sigma }=\sigma \cdot \left(1+\varepsilon \right)$$

**Figure 3:**
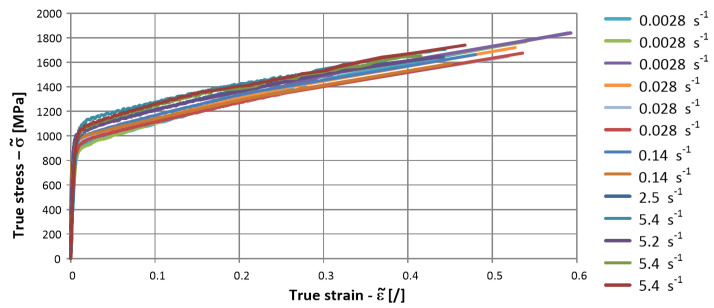
True stress-true strain curves for low strain-rate tensile tests.

ε and σ were the corresponding engineering strain and stress, respectively. The obtained true stress–true strain curves are shown in Figure 3 and represent a basis for the applied material model at quasi-static conditions.

The obtained true stress–true strain curves for lower strain rates (below 0.14 s^−1^) were transformed using Equations (1) and (2) until necking. The parts of the curve after the necking onset were obtained with a connection between the point of ultimate tensile stress and the point, which was calculated using the measurement of the final specimen cross-section and force right before the fracture.

**Figure 4:**
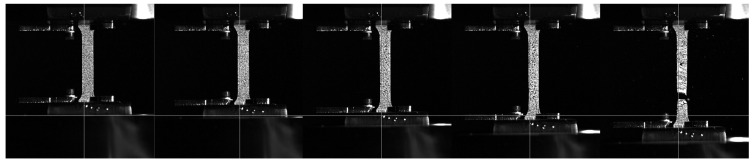
A sequence of recorded tensile tests with high-speed camera.

For higher strain rates, the true stress–true strain curves were obtained through the digital image correlation (DIC) method and the high-speed camera recordings—see Figure 4. During the tensile test, the high-speed camera frame rate and data acquisition were synchronised; therefore, the load data were recorded for each frame. A resolution was set to 800 × 1080 pixels, and the frame rate was 4096 frames per second. By using a tracking algorithm in the software Xcitex ProAnalyst 2023 (https://www.xcitex.com/products/proanalyst/, accessed on 30 April 2024), measurements of the specimen width were obtained for each recorded time frame during the whole experiment. 

A product of the measured width and the calculated thickness results in an area of the specimen at each time instance of the experiment [18,19]. For this purpose, a presumption of isotropic material response was adopted. Along with the measured force, the true stress and true strain were calculated as follows:

(3) $$\tilde{\varepsilon }=\ln\left(\frac{A_{0}}{A}\right)$$

(4) $$\tilde{\sigma }=\frac{F}{A}$$

*A*_0_ is an initial cross-section, and *A* and *F* are cross-section and measured force in recorded time frame, respectively. 

**Figure 5:**
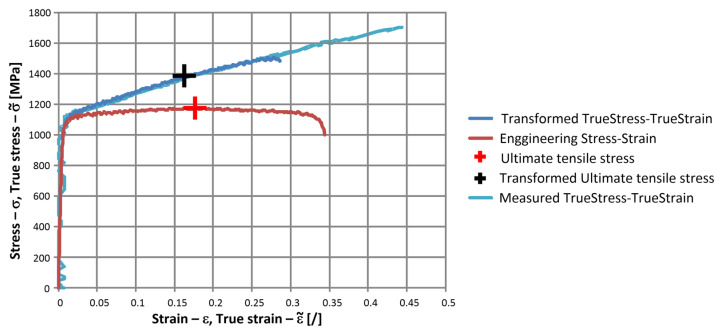
Comparison of engineering stress–engineering strain curve and the transformed true stress–true strain curve.

In Figure 5, the engineering stress–strain for the strain rate of 5.4 s^−1^ curve is presented together with its transformed true stress–true strain curve. By comparing the transformed true stress–true strain curve and the measured true stress–true strain curve, it can be concluded that the material characteristic continues with an almost linear shape after the necking occurs until the fracture of the specimen. The stress and strain at this point were calculated using the final specimen’s cross-section and loading force just before the fracture occurred, which coincided with the end of the direct true stress–true strain curve. 

Without the high-speed camera recordings, the measurement of the width and thickness of the specimen would be impossible due to the very short duration of the experiment, which was less than 1 s. Therefore, we could not use the conventional method of direct measurement of the specimen’s cross-section area to obtain the true stress and true strain data. In the material model, which was used in this research, the piecewise linear curve should be defined. 

Due to similar curve shapes for lower and higher strain rates, it was decided to use the whole curve in the definition of material model.

### 2.2. Experimental Determination of Material Behaviour at High Strain Rates

The main objective was to determine the strain-rate dependent material parameters for simulating the behaviour of a high-strength steel sheet metal. To study the dynamic behaviour of thin flat metal sheets, a similar experimental arrangement was designed according to the ASTM D5420 standard [20], which describes a test method for measuring the impact resistance of a flat plastic specimen subjected to a falling weight. 

**Figure 6:**
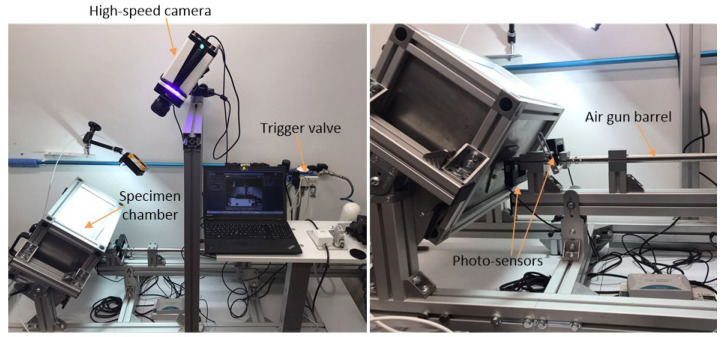
The experimental setup with an air gun and high-speed camera.

In our experimental setup, a steel ball with a diameter of 13.6 mm and a weight of 10.3 g was shot to a flat specimen at different angles with different velocities. The experimental setup for shooting the steel ball into a deformable plate is shown in Figure 6.

**Figure 7:**
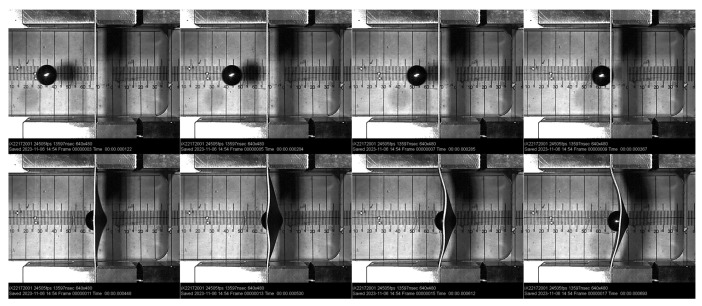
Sequence of high-velocity ball impact to metal sheet.

The ball velocities were measured with two photo-diode sensors mounted on the last part of the air gun barrel. The distance between the photo sensors is 120 mm, and the time from the first to the second sensor is recorded to obtain the ball velocity at the end of the air gun barrel. These measurements were also verified through high-speed camera recordings (see Figure 7) because some acceleration of the ball in the barrel remains after the ball passes the muzzle brake. For this purpose, the i-SPEED 508 high-speed camera from IX Cameras was used. After analysing all the recordings, the correction of the measured velocities from the photo sensors was done, and the real values of the ball velocities were considered for the testing conditions. 

Due to the limitations of the air gun mounted to the experimental setup, the lowest striking velocity was between 100 and 115 m/s, and the maximum velocity was up to 170 m/s. The specimens were metal sheet plates with dimensions of 98 mm × 60 mm and 1 mm of thickness. They were fixed along the shorter sides; therefore, the free area of the specimen was 60 × 60 mm². The testing conditions are presented in Table 2.

**Figure 8:**
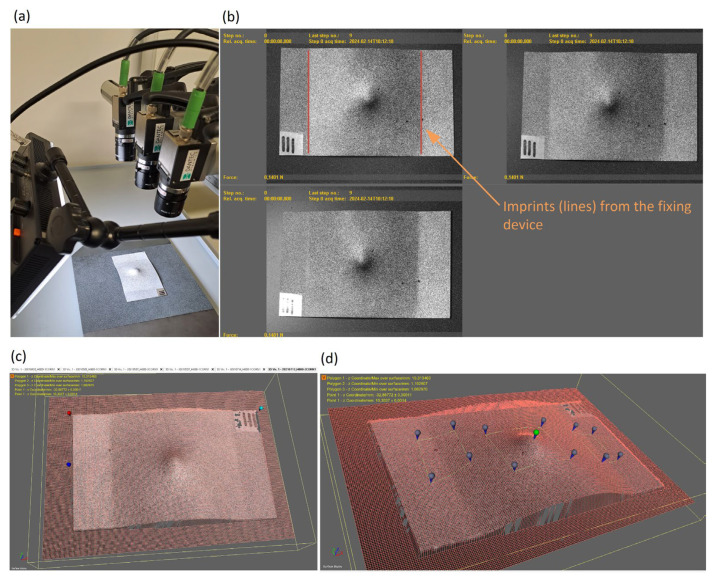
The 3D digital image correlation (DIC) system with three cameras (**a**), three images of the deformed specimens (**b**), the definition of the coordinate system (red, green and blue points) (**c**), marking of points (blue) in the regions of interest (**d**).

The gross geometric measurements were obtained using a digital image correlation (DIC) method, as described in the continuation. A random pattern with acrylic paint (white base colour with black dots) was applied to the test subject. In addition, an even larger flat plate was prepared, which served as a base for the test subjects. A random colour pattern was also applied to this base: a black base colour with white dots was used so that the contrast in the photos between the test subject and the base was slightly higher. The Dantec 3D DIC system with three 5 MPx cameras and an LED lighting module was directed towards the base on which the subject was placed; see Figure 8a.

The distance of the cameras from the object was minimal, so the entire resolution of the sensor was used as much as possible, thereby improving the resolution of the photos and, later, the accuracy of the calculated values in the DIC program. Before capturing the photos, a 3D calibration of the system position (i.e., cameras and focal lengths) was carried out, which is necessary for image correlation. Each subject was photographed with an image acquisition frequency of 10 Hz, and 10 photographs were saved. The captured images were then processed using the Dantec Istra4D software. Correlation of the images enables the drawing of the subject’s contour. The density of the calculation points for the image correlation was chosen so that considered contours were reliably detected.

To determine the height of the deformed sheet metal within the selected region of interest, a starting coordinate system was first determined; see Figure 8c. The X–Y plane of the coordinate system was placed on the lower base on which the test subject was lying. In the image processing software, the local field around the top of the deformed part was marked, and the maximum value from the coordinate in this field was indicated. Similar fields were also marked on the lines (i.e., slight imprints on the specimen surface); see Figure 8b, where the test subject was fixed to the housing during the shooting experiment. The minimum values of the coordinates on these lines were pointed out; see Figure 8d. Then, the average of the minimum values on the left and right sides of the specimen was calculated. Finally, by considering the thickness of the test specimen and the maximum detected height, the actual indentation depth of the imprint in the sheet metal was determined.

### 2.3. Identification of the Strain-Rate-Dependent Material Parameters

A strain rate dependency of the yield stress $\sigma_{Y}\left(\varepsilon_{pl}\right)$ on the strain rate $\varepsilon_{pl}$ was modelled with a Cowper–Symonds model. According to the applied model, the static yield curve $\sigma_{Y}\left(\varepsilon_{pl}\right)$ is shifted vertically as a function of the plastic strain rate ${\dot{\varepsilon }}_{pl}$ as follows [1,2]:

(5) $$\sigma_{Y}\left(\varepsilon_{pl}\right)=\sigma_{Y,S}\left(\varepsilon_{pl}\right)+SIGY\cdot {\left(\frac{{\dot{\varepsilon }}_{pl}}{C}\right)}^{1/p}$$

*SIGY* should represent the yield stress at quasi-static conditions; *C* and *p* are Cowper–Symonds parameters. To describe the material’s response to different strain rates, these three parameters need to be estimated. A two-phase procedure was applied for this purpose:

- A rough estimation of the three parameters *SIGY*, *C*, and *p* was first conducted with a grid-search method for the tensile tests at low strain rates.
- After the first phase, the *SIGY* parameter was fixed, and the two parameters, *C* and *p*, were fine-tuned using a reverse-engineering approach combined with a genetic algorithm optimisation procedure in the second phase.

#### 2.3.1. PHASE 1—Rough Estimation on the Basis of the Tensile Tests at a Low Strain Rate

The three parameters *SIGY*, *C* and *p* were first divided according to Table 3. Then, the measured true stress–true strain plastic curves were shifted vertically for their *R*_*p*0.2_ value; i.e., for each plastic flow curve, the knee point of the curve was shifted to zero. The average value of the shifted curves represented the curve $\sigma_{Y}\left(\varepsilon_{pl}\right)$ in Equation (5).

**Figure 9:**
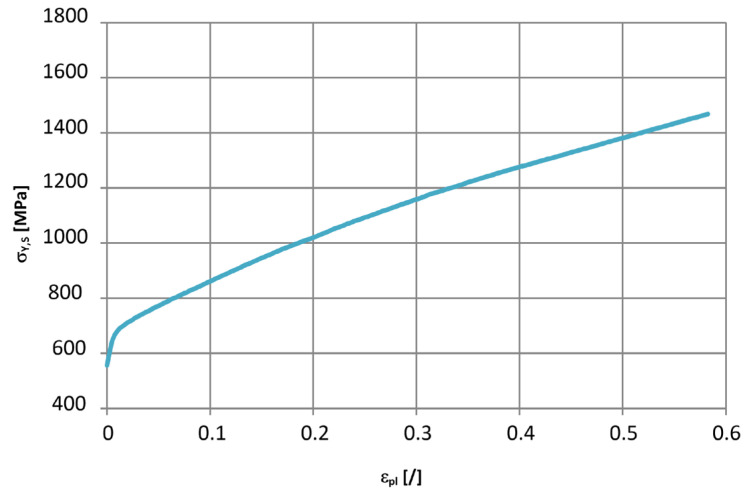
Used flow curve $\sigma_{Y,S}\left(\varepsilon_{pl}\right)$ at quasi-static strain rates.

Now, a special note needs to be added here. In Equation (5), the *SIGY* parameter should be the yield stress in quasi-static loading. This means that the strain rate for quasi-static loading needs to be defined first. It was set to a value of 10^−10^ s^−1^ in our case. Then, the corresponding flow curve $\sigma_{Y,S}\left(\varepsilon_{pl}\right)$ needs to be defined. We decided to model it with a piecewise linear function, which started below the knee point of the measured true stress–true strain curves—see Figure 9. This means that the *SIGY* parameter becomes only a parameter that needs to be estimated and is not directly related to the material’s *R_p_*_0.2_ value. Moreover, we have discovered that Equation (5) models the material response very poorly if the *SIGY* parameter is set to the value of *R_p_*_0.2_ for the smallest strain rate measured. Since there was no clear point of deviation from linearity in the case of the high-strain rate experiments, an inverse analysis was followed in the second phase in order to obtain the final estimates of the three parameters from Equation (5).

For every combination of the three parameters *SIGY*, *C*, and *p* in Table 3, $\sigma_{Y,S}\left(\varepsilon_{pl}\right)$ was modelled with Equation (5) for every tensile test at low strain rates. Then, a sum of the squared distances (*SSQD*) cost function was calculated for each of the measured and modelled yield curves. Finally, the *SSQD* cost functions for the individual yield curves were added together to obtain the final value of the combined cost function over all the low strain rates *SSQD*_LSR_:

(6) $${SSQD}_{LSR}=\sum_{LSR\_cases}\left[\sum_{\varepsilon \_domain}{\left(\sigma_{Y}\left(\varepsilon_{pl}\right)-{\hat{\sigma }}_{Y}\left(\varepsilon_{pl}\right)\right)}^{2}\right]$$

The smallest value of the summarised cost function *SSQD*_LSR_ was obtained for the following combination of the Cowper-Symonds parameters: *SIGY* = 400 MPa, *C* = 50 and *p* = 25. At this point, the value *SIGY* = 400 MPa was fixed, and the estimations of the two parameters, C and p, were refined. Again, the grid search algorithm was used to minimise the combined cost function from Equation (6), but now, it has a different division of the two parameters; see Table 4.

**Figure 10:**
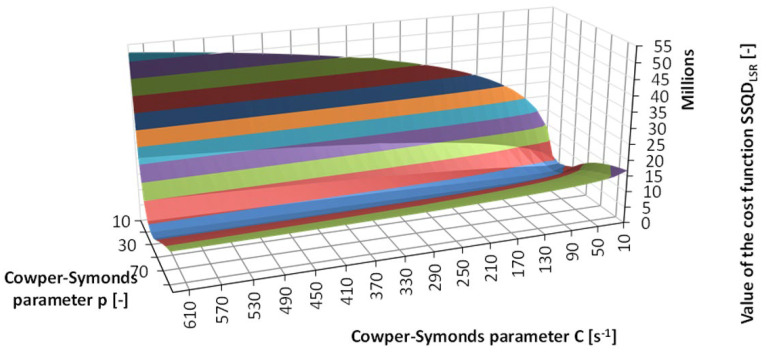
Surface of the summarised cost function *SSQD*_LSR_ in Phase 1.

The *SSQD*_LSR_ cost function from Equation (6) was calculated for each combination of the parameters C and p in Table 3. The *SIGY* parameter was kept constant at 400 MPa. The surface of the summarised cost function *SSQD*_LSR_ is presented in Figure 10. Now, the most optimal estimates of the two parameters *C* and *p* were: *C* = 210 and *p* = 30.

#### 2.3.2. PHASE 2—Enhanced Estimation Based on the Reverse-Engineering Approach

**Figure 11:**
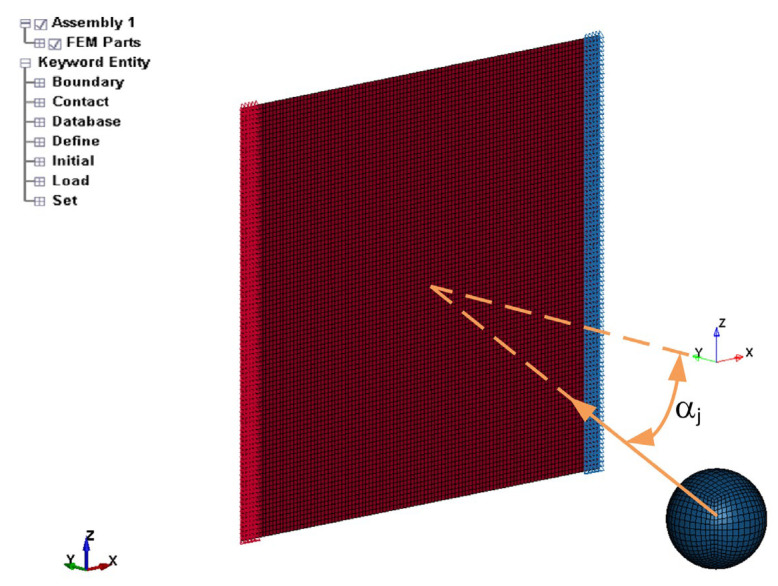
Finite element (FE) model in LS-Dyna software.

To consider high strain rate experiments for estimating the Cowper–Symonds parameters *C* and *p*, a series of explicit dynamic finite element (FE) simulations were carried out using LS-DYNA R13 software (https://lsdyna.ansys.com/, accessed on 30 April 2024). The FE model consisted of the plate-like specimen and the flying ball; see Figure 11.

The steel-sheet model had 9984 four-node shell finite elements with a Hughes–Liu co-rotational formulation. The mesh density is constant throughout the model in order to simulate the indentation accurately. The rigid ball is modelled with 19,208 eight-node constant-stress solid finite elements. In the finite element model, the nodes on both sides of the thin sheet had all their degrees of freedom fixed; see Figure 11. An initial velocity vector was prescribed to the ball to input the ball’s velocity and travelling direction. A rigid ball was fired into the centre of the sheet at three different angles (0°, 20°, 35°) with different values of the velocity, which are recorded in Table 2. Between the steel sheet and the rigid ball, there was an AUTO_SURFACE_TO_SURFACE contact with a friction coefficient *µ* = 0.2 [4]. The numerical simulations were carried out on an HPC computer with 1024 processing nodes with 24 processors at each node. For this reason, mass scaling was not used in the finite element simulations in order to diminish the processing time. During each simulation, the gross geometric dimensions of the specimen were recorded for further processing.

**Figure 12:**
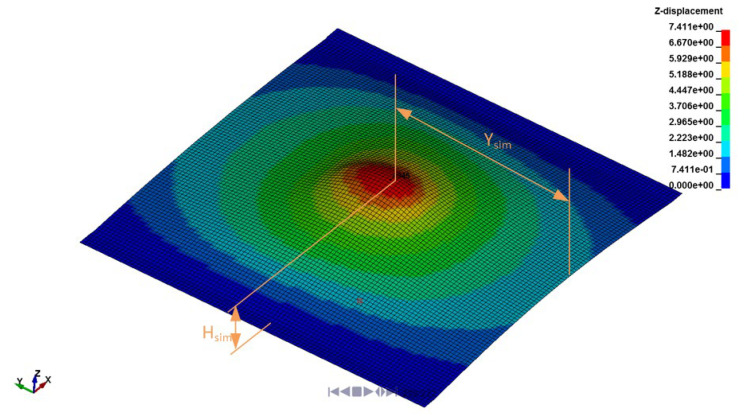
Results of nodal displacement in Z-direction.

The ball was modelled with a rigid material MAT_RIGID (MAT_20). For the plate, the MAT_PIECEWISE_LINEAR_PLASTICITY (MAT_24) material model with a Von Mises plasticity and a kinematic hardening rule in LS-Dyna package software [1,2] was used in the form of Equation (5). This means that the applied method for estimating the missing strain-rate-dependent parameters did not influence the hardening model and the yield model. For the material parameters, the same static flow curve $\sigma_{Y}\left(\varepsilon_{pl}\right)$ and the *SIGY* parameter were used as in Phase 1. In Phase 2, the two Cowper–Symonds parameters, *C* and *p*, were divided into 7 equidistant values around the best parameter values from Phase 1; see Table 5. For each combination of the material parameters from Table 4, nine (9) explicit dynamic simulations were performed to model the 9 experiments from Table 2. Altogether, 441 FE simulation runs were performed. An example of the explicit dynamic simulation is presented in Figure 12.

To estimate optimal values of the Cowper–Symonds material parameters for a wide range of strain rates (from quasi-static to high strain rates), the optimisation process has to be performed in such a way that results from static and dynamic experiments could be included.

A multi-criteria optimisation process was used to identify the optimal combination of the applied material model parameters. For the low-strain-rate experiments, the summarised cost function *SSQD*_LSR_ from Equation (6) was applied. It was calculated for each combination of the material parameters from Table 4. For the high-strain-rate experiments, the cost function was calculated as follows:

Estimating the indentation depth *H_ij_*_,sim_ and the coordinate of the maximum indentation *Y*_ij,sim_ from the simulated results for each boundary/initial condition *j* = 1, …, 9 and each combination of the material parameters *i* = 1, …, *n*; *n* = 49.

Calculating the cost function *SSQD*_HSR,*i*_ for the high strain-rate experiments for each combination of the material parameters from Table 4:

(7) $${SSQD}_{HSR,i}=\sum_{j=1}^{12}\left[0.5\cdot {\left(H_{ij,sim}-H_{j,exp}\right)}^{2}+0.5\cdot {\left(Y_{ij,sim}-Y_{j,exp}\right)}^{2}\right]$$

Calculating the multi-criteria cost function *CF_i_* for low- and high-strain-rate experiments:

(8) $${CF}_{i}=u\cdot \frac{{SSQD}_{LSR,i}}{1000000}+(1-u)\cdot {SSQD}_{HSR,i}$$

The low-strain-rate cost function *SSQD*_LSR_ from Equation (6) was divided by one million to obtain the same order of magnitude as the high-strain-rate cost function *SSQD*_HSR_ from Equation (7). A sensitivity analysis for the weight *u* was carried out, in which this weight was varied between 0.2 and 0.8. It turned out that the results of the optimisation process were the same if this weight was between 0.3 and 0.7. For this reason, the final value of 0.5 was selected for the weight *u*.

Before the optimisation process was carried out, a response surface was determined for the *n* = 49 calculated values of the cost function *CF_i_*. The adopted form of the cost function was taken from the work of Škrlec and Klemenc [4]. The global trend of the cost function *CF_i_* was first approximated with a polynomial of the third order:

(9) $$\hat{CF}=\hat{CF}\left(C,p\right)=a_{0}+a_{1}\cdot C+a_{2}\cdot p+a_{3}\cdot C^{2}+a_{4}\cdot C\cdot p+a_{5}\cdot C^{2}+\;+a_{6}\cdot C^{3}+a_{7}\cdot C^{2}\cdot p+a_{8}\cdot C\cdot p^{2}+a_{9}\cdot p^{3}$$

Then, a residuum *Res_i_* was calculated for each combination of the material parameters *C* and *p*:

(10) $${\;\;Res}_{i}={Res}_{i}\left(C_{i},p_{i}\right)=CF\left(C_{i},p_{i}\right)-\hat{CF}\left(C_{i},p_{i}\right)\;\;\;;\;\;\;i=1,\ldots ,n\;\;\;;\;\;n=49$$

The residues *Res_i_* were finally interpolated using the sum of the weighted two-dimensional Gaussian functions with diagonal covariance matrices **S***_i_*:

(11) x=(C,p)

(12) $$Res\left(C,p\right)=Res(x)=\sum_{i=1}^{n}b_{i}\cdot w(x-x_{i},S_{i})$$

(13) $$w\left(x-x_{i},S_{i}\right)={\left(2\cdot \pi \right)}^{-1}\cdot {det(S_{i})}^{-1/2}\cdot exp\left[\frac{1}{2}\cdot {\left(x-x_{i}\right)}^{T}\cdot S_{i}^{-1}\cdot (x-x_{i})\right]$$

The diagonal elements of the covariance matrices **S***_i_* are equal to the squared values of the half-distances to the nearest neighbour in (*C*, *p*) space along the *C* or *p* axis. The weighting parameters *b_i_* in Equation (12) were calculated with the following matrix equation:

(14) $$b=W^{-1}\cdot r$$

(15) $$b={\left[b_{1},b_{2},\ldots ,b_{i},\ldots ,b_{n}\right]}^{T}$$

(16) $$W=\left[\begin{array}{ccc} w(0,S_{1}) & \cdots & w(x_{1}-x_{n},S_{n})\\ \vdots & \ddots & \vdots \\ w(x_{n}-x_{n},S_{n}) & \cdots & w(0,S_{n}) \end{array}\right]$$

(17) $$r={\left[{Res}_{1},Res,\ldots ,{Res}_{i},\ldots ,{Res}_{n}\right]}^{T}$$

In this manner, an exact interpolation function with a well-expressed global trend was obtained for the discrete values of the cost function *CF* in Equation (8):

(18) $$CF=CF\left(C,p\right)=CF\left(x\right)=\hat{CF}\left(x\right)+Res(x)$$

For the cost function in Equation (18), a minimum was determined with a real-valued genetic algorithm (RVGA). The searching domains for the two Cowper–Symonds parameters were as follows: *C* ∈ [70, 550] and *p* ∈ [15, 40]. The number of genetic algorithm iterations was 10,000. The RVGA code was programmed by the authors of this paper. Theoretical details can be found in Klemenc and Fajdiga [21].

## 3. Results

The obtained true stress–true strain curves for the low strain rate tensile tests are presented in Figure 3 in Section 2.1. The indentation depths and Y-coordinates of the maximum depths, which were determined according to the procedure described in Section 2.2, are presented in Table 6.

From the results in Table 6, it is clear that the scatter of the experimental results is relatively small, which means that the experimental arrangement was appropriate for the study. Furthermore, we can see that the indentation depth increases with the increasing velocity. Another well-observed characteristic is the point with the deepest indentation depth, i.e., the Y-coordinate of the maximum indentation depth. This coordinate changes with varying impact angles, which implies different impact dynamics in the case when the impact direction of the ball is not perpendicular to the plate specimen. The strain rates for the impact ball testing were determined on the basis of simulations and were up to 400 s^−1^ for the highest ball velocity. These strain rates could not be measured directly but were determined from the results of numerical simulations for the most optimal values of the Cowper–Symonds parameters. 

The finite element simulations of the experimental arrangement were performed on the HPC computer Prelog at the University of Ljubljana, Faculty of Mechanical Engineering. The time spent for one numerical simulation at two processing cores with 24 processors was 58 min. If the calculation time step of the simulation was reduced below reasonable values so that the termination time could not be reached, the time limit was triggered, and the simulation calculation was automatically interrupted. The final gross geometric data of the interrupted simulations were set to maximum values to include these results in the optimisation process.

**Figure 13:**
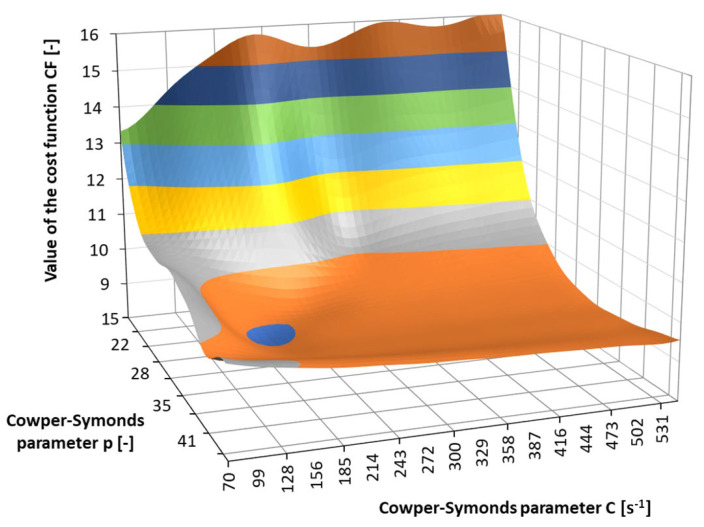
Interpolated cost function *CF* for the low- and high-strain-rate experiments.

**Figure 14:**
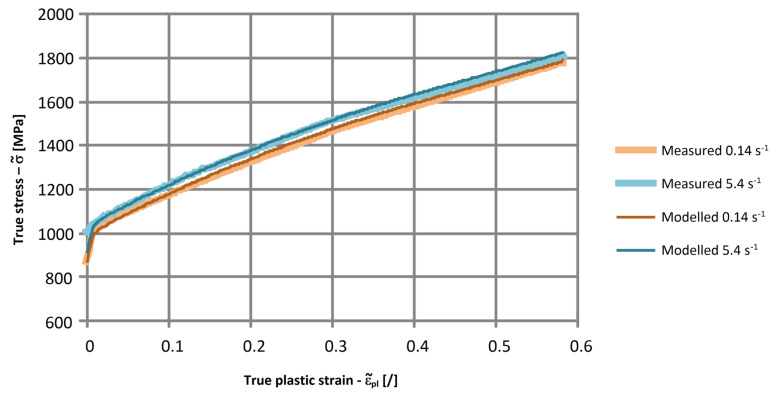
Comparison of the modelled and measured yield curves for the tensile tests.

The interpolated cost function *CF* from Equation (18) for the low-strain-rate results in Figure 3 and the high-strain-rate results in Table 6 are presented in Figure 13. For the cost function in Figure 13, the optimal values of the Cowper–Symonds parameters *C* and *p* are *C* = 172.4 s^−1^ and *p* = 30.4. In Figure 14, an agreement between the selected modelled and measured yield curves from Section 2.1 is presented. For the modelled curves, the value of *SIGY* = 400 MPa was considered. It can be concluded that the modelled and experimentally determined yield curves are in very good agreement. The agreement between the results of numerical simulations and measured deformations of the plate after the ball impact is good. A relative error between 0.8% and 8.4% was obtained for the *Y*-coordinate of the maximum indentation depth, and a relative error between 0.7% and 22.5% was obtained for the maximum indentation depth.

## 4. Discussion

In Table 7, optimal values of the parameters *p* and *C* for the SZBS800 steel are compared to the Cowper–Symonds parameters for similar materials from the literature.

If the value of the parameter *p* is compared to the values from the literature, it is clear that, in our case, the value is approximately 10 times higher than all other values. In contrast, our estimate of the parameter *C* is the lowest compared to those from the literature, with only one exception. However, this does not imply that the estimated values from our optimisation are false. It is clear from Figure 12 that the response surface in the domain where the optimal value was found is extremely flat and wide. Consequently, the interval for the optimum candidates spans over multiple orders of magnitude for both parameters, especially for parameter *C*. Moreover, it was discovered in the sensitivity analysis that changing the weight *u* in Equation (8) does not result in significantly different estimates of the parameters *p* and *C*.

From the comparison, it is clear that strain rate parameter values depended strongly on the testing method. Namely, the chosen testing method and strain rates achieved during experimental procedures have a significant influence. For the parameter *C*, values up to 10^7^ s^−1^ can be found in the literature if there are high strain rates in the interval above 10^3^ s^−1^. In contrast, the values of parameter *C* are six orders of magnitude lower if there are strain rates only up to 0.1 s^−1^. Moreover, the two parameters, p and C, are strongly correlated. This is a consequence of the shape of the cost function. We have discovered before [4] that many different pairs of values *p* and *C* result in similar material responses, especially in the high-strain-rate domain. For this reason, it is very important to estimate the strain-rate-dependent material parameters over a wide range of strain rates. Finally, the estimated values of the parameters *p* and *C* also depend on the formulation of the Cowper–Symonds model. In our case, the visco-plastic formulation was used in Equation (5) to allow for parallel transformation of the yield curve along the stress axis. If another formulation of the Cowper–Symonds model was used (i.e., with scaling of the whole yield curve as a function of the strain rate; see [1,2]), different values of the parameters *p* and *C* would be obtained. At this point, we should also mention that the optimal values of the parameters *p* and *C* depend on the selected value of the *SIGY* parameter. However, to model the material response as well as possible, it is important that the complete flow curve from Equation (5) generalises the material response at low and high strain rates. Even though this means that the parameter *SIGY* does not reflect the *R*_*p*0.2_ value from the experiments.

## 5. Conclusions

In this article, the response of the SZBS800 high-strength steel to different loading rates was experimentally determined. The strain-rate-dependent parameters of the Cowper–Symonds material model were estimated on the basis of a standard tensile test at different loading rates. In this manner, the material response up to strain rates of 5.4 s^−1^ was obtained. The true stress–true strain curves were obtained based on transformation equations and directly measured using high-speed camera recordings with full HD resolution and the digital image correlation method. Both procedures gave very similar curve shapes for the whole range of strains up to fracture. 

The material response at higher strain rates was investigated using an unconventional experiment in which the steel ball was shot to a deformable flat specimen. Using a reversed engineering approach, a combination of numerical simulations, multi-criteria cost function, response surface, and numerical optimisation scheme was used to estimate the parameters of the Cowper–Symonds material model. The main conclusions are the following:

- The estimated values of the Cowper–Symonds parameters *p* and *C* differ somewhat from the data in the literature for similar materials.
- Parameter estimates are strongly dependent on the applied testing methods and strain rates.
- Due to the shape of the multi-criteria cost function, a wide domain of the combinations for the material parameters *p* and *C* yield acceptable results.
- Estimated values of the material parameters also depend on the mathematical formulation for the strain-rate effects.

## Figures and Tables

**Table 1:** Chemical composition of SZBS800 steel [16].

C	Si	Mn	P	S	Al	B	Cu
max.	max.	max.	max.	max.	min.	max.	max.
0.18%	1.00%	2.20%	0.05%	0.01%	0.015–1.2%	0.005%	0.2%

In addition, the elements Nb, V, and Ti are each alloyed either individually or in combination (Nb + V + Ti ≤ 0.25%).

**Table 2:** Testing conditions for the impact ball testing.

Specimen Number	Impact Angle *a_j_* [°]	Ball Velocity *v_j_* [m/s]
20210503_H800-1	0	111.5
20231108_H800-1	0	133.3
20210504_H800-1	0	155.3
20231108_H800-3	20	111.2
20231108_H800-2	20	132.6
20210714_H800-1	20	154.2
20210507_H800-2	35	113.1
20231110_H800-1	35	131.9
20210713_H800-1	35	153.5

**Table 3:** Primary division of the parameters *SIGY*, *C*, and *p*.

*SIGY* [MPa]	*C* [s^−1^]	*p* [/]
200	1	0.1
300	2.5	0.25
400	5	0.5
500	10	1
600	50	2.5
	100	5
	250	10
	500	15
	2500	25
	10,000	50
		100

**Table 4:** Secondary division of the parameters *C* and *p*.

*p* [/]	*C* [s^−1^]	*p* [/]	*C* [s^−1^]
10	10	80	190
15	30	90	210
20	50	100	230
25	70		250
30	90		270
40	110		300
50	130		600
60	150		1000
70	170		1500

**Table 5:** Division of the parameters *C* and *p* for the LS-DYNA simulations.

*p* [/]	*C* [s^−1^]	*p* [/]	*C* [s^−1^]
15	70	35	390
20	150	40	470
25	230	45	550
30	310		

**Table 6:** Results of impact ball testing.

Specimen Number	Impact Angle *α_j_* [°]	Ball Velocity *v_j_* [m/s]	Y-Coordinate of max. Indentation *Y_j,_*_exp_ [mm]	Maximum Indentation Depth *H_j,_*_exp_ [mm]
20210503_H800-1	0	111.5	29.762	5.768
20231108_H800-1	0	133.3	29.282	8.057
20210504_H800-1	0	155.3	29.716	8.497
20231108_H800-3	20	111.2	30.306	5.975
20231108_H800-2	20	132.6	29.575	6.940
20210714_H800-1	20	154.2	32.632	8.025
20210507_H800-2	35	113.1	32.325	5.726
20231110_H800-1	35	131.9	32.331	6.055
20210713_H800-1	35	153.5	33.821	6.911

**Table 7:** Comparison with the Cowper–Symonds parameters *C* and *p* for materials from the literature.

Material Name	EN Code	Testing Method	*p* [-]	*C* [s^−1^]
S690QL ultra-high-strength steel—EN 10025 [22]	1.8931	Low strain rates 10^−3^ s^−1^: Zwick/Roell—Z50 (ZwickRoell GmbH & Co., Ulm, Germany) Medium strain rates 3 s^−1^–30 s^−1^: Hydro-pneumatic machine; High strain rates 250 s^−1^–950 s^−1^: Split Hopkinson Tensile Bar	2.3 to 4.49	121,783 to 212,352
Hot-forming steel 22MnB5 [23]	1.5528	Low strain rates 10^−3^ s^−1^: WDW-100E uniaxial tensile testing machine; High strain rates 2000 s^−1^–4000 s^−1^: Split Hopkinson Pressure Bar	3.2050	6277.8
S690 high-strength structural steel [11]	1.3964	Low strain rates 10^−3^ s^−1^: universal electromechanical testing machine; Intermediate strain rates 10 s^−1^–200 s^−1^: High-speed tensile testing machine	6.1–6.7	1.2 × 10^7^ to 3.3 × 10^7^
high-strength reinforcing steel HTRB600E [24]		Low strain rates: below 2 × 10^−3^ s^−1^: electromechanical universal testing machine; Elevated strain rates 0.018–585 s^−1^: high-speed servo-hydraulic tensile testing machine Zwick/Roell HTM5020	5.925 to 6.027	1.83 × 10^7^ to 8.67 × 10^7^
Q390D steel [5]		Low strain rates 8.4 × 10^−4^ s^−1^: INSTRON 5569 uniaxial tensile testing machine (Instron, Norwood, MA, USA); High strain rates 837.1 s^−1^–3368.1 s^−1^: Split Hopkinson pressure Bar	1.31 to 2.4	3097.14 to 3861.44
Duplex Stainless Steel 2507 [6]	1.4410	Low strain rates 0.0001–0.1: electromechanical universal testing machine (Zwick/Roell Z250)	1.7566	2.68274
MP800HY Steel [25]		Low strain rates 10^−3^ s^−1^: Zwick/Roell—Z50; Medium strain rates 5 s^−1^–25 s^−1^: Hydro-pneumatic machine; High strain rates 250 s^−1^–750 s^−1^: modified Hopkinson Bar	2.2146	32,338
steel B500A [26]		Low strain rates 10^−4^ s^−1^: universal electromechanical testing machine; High strain rates 250 s^−1^–1000 s^−1^: Split Hopkinson Tensile Bar	1.654 to 4.4677	5.574 to 525.444
Steel HRB500E [27]		Low strain rates 2.5 × 10^−4^–5.3 × 10^−1^ s^−1^: universal electromechanical testing machine; High strain rates 0.1 s^−1^–550 s^−1^: Zwick/Roell HTM5020 servo-hydraulic high-speed testing machine	4.906	264,713
SZBS800 steel	1.0998	Low strain rates: 100 kN MTS Landmark testing machine; High strain rates: shooting ball into flat specimen	30.4	172.4

## Data Availability

The data presented in this study are available on request from the corresponding author. The data are not publicly available due to privacy reason.
